# Delirium Risk Score in Elderly Patients with Cervical Spinal Cord Injury and/or Cervical Fracture

**DOI:** 10.3390/jcm12062387

**Published:** 2023-03-20

**Authors:** Koji Tamai, Hidetomi Terai, Hiroaki Nakamura, Noriaki Yokogawa, Takeshi Sasagawa, Hiroaki Nakashima, Naoki Segi, Sadayuki Ito, Toru Funayama, Fumihiko Eto, Akihiro Yamaji, Kota Watanabe, Junichi Yamane, Kazuki Takeda, Takeo Furuya, Atsushi Yunde, Hideaki Nakajima, Tomohiro Yamada, Tomohiko Hasegawa, Yoshinori Terashima, Ryosuke Hirota, Hidenori Suzuki, Yasuaki Imajo, Shota Ikegami, Masashi Uehara, Hitoshi Tonomura, Munehiro Sakata, Ko Hashimoto, Yoshito Onoda, Kenichi Kawaguchi, Yohei Haruta, Nobuyuki Suzuki, Kenji Kato, Hiroshi Uei, Hirokatsu Sawada, Kazuo Nakanishi, Kosuke Misaki, Akiyoshi Kuroda, Gen Inoue, Kenichiro Kakutani, Yuji Kakiuchi, Katsuhito Kiyasu, Hiroyuki Tominaga, Hiroto Tokumoto, Yoichi Iizuka, Eiji Takasawa, Koji Akeda, Norihiko Takegami, Haruki Funao, Yasushi Oshima, Takashi Kaito, Daisuke Sakai, Toshitaka Yoshii, Tetsuro Ohba, Bungo Otsuki, Shoji Seki, Masashi Miyazaki, Masayuki Ishihara, Seiji Okada, Shiro Imagama, Satoshi Kato

**Affiliations:** 1 Department of Orthopaedic Surgery, Osaka Metropolitan University Graduate School of Medicine, 1-4-3 Asahimachi, Abeno-ku, Osaka-City, Osaka 545-8585, Japan; 2 Department of Orthopaedic Surgery, Graduate School of Medical Sciences, Kanazawa University, 13-1 Takara-machi, Kanazawa, Ishikawa 920-8641, Japan; 3 Department of Orthopedics Surgery, Toyama Prefectural Central Hospital, 2-2-78 Nishinagae, Toyama, Toyama 930-8550, Japan; 4 Department of Orthopedic Surgery, Nagoya University Graduate School of Medicine, Nagoya, 65 Tsurumai-cho, Showa-ku, Nagoya 466-8550, Japan; 5 Department of Orthopaedic Surgery, Faculty of Medicine, University of Tsukuba, 1-1-1 Tennodai, Tsukuba, Ibaraki 305-8575, Japan; 6 Department of Orthopaedic Surgery, Graduate School of Comprehensive Human Sciences, University of Tsukuba, 1-1-1 Tennodai, Tsukuba, Ibaraki 305-8575, Japan; 7 Department of Orthopaedic Surgery, Ibaraki Seinan Medical Center Hospital, 2190, Sakaimachi, Sashima, Ibaraki 306-0433, Japan; 8 Department of Orthopaedic Surgery, Keio University School of Medicine, 35 Shinanomachi, Shinjuku-ku, Tokyo 160-8582, Japan; 9 Department of Orthopaedic Surgery, Japanese Red Cross Shizuoka Hospital, 8-2 Otemachi, Aoi-ku, Shizuoka 420-0853, Japan; 10 Department of Orthopaedic Surgery, Graduate School of Medicine, Chiba University, 1-8-1 Inohana, Chuo-ku, Chiba, Chiba 260-8670, Japan; 11 Department of Orthopaedics and Rehabilitation Medicine, Faculty of Medical Sciences University of Fukui, 23-3 Matsuoka Shimoaizuki, Eiheiji-cho, Yoshida-gun, Fukui 910-1193, Japan; 12 Department of Orthopaedic Surgery, Hamamatsu University School of Medicine, 1-20-1, Handayama, Higashi-ku, Hamamatsu City, Shizuoka 431-3192, Japan; 13 Department of Orthopaedic Surgery, Nagoya Kyoritsu Hospital, 1-172 Hokke, Nakagawa-ku, Nagoya-shi, Aichi 454-0933, Japan; 14 Department of Orthopaedic Surgery, Sapporo Medical University, South 1-West 16-291, Chuo-ku, Sapporo 060-8543, Japan; 15 Department of Orthopaedic Surgery, Matsuda Orthopedic Memorial Hospital, North 18-East 4-1 Kita-ku, Sapporo 001-0018, Japan; 16 Department of Orthopaedic Surgery, Yamaguchi University Graduate School of Medicine, 1-1-1 Minami-Kogushi, Ube City, Yamaguchi 755-8505, Japan; 17 Department of Orthopaedic Surgery, Shinshu University School of Medicine, 3-1-1 Asahi, Matsumoto, Nagano 390-8621, Japan; 18 Department of Orthopaedics, Graduate School of Medical Science, Kyoto Prefectural University of Medicine, Kawaramachi-Hirokoji, Kamigyo-ku, Kyoto 602-8566, Japan; 19 Department of Orthopaedics, Saiseikai Shiga Hospital, 2-4-1 Ohashi Ritto, Shiga 520-3046, Japan; 20 Department of Orthopaedic Surgery, Tohoku University Graduate School of Medicine, 1-1 Seiryo-machi, Aoba-ku, Sendai, Miyagi 980-8574, Japan; 21 Department of Orthopaedic Surgery, Graduate School of Medical Sciences, Kyushu University, 3-1-1 Maidashi Higashi-ku, Fukuoka 812-8582, Japan; 22 Department of Orthopaedic Surgery, Nagoya City University Graduate School of Medical Sciences, 1 Kawasumi, Mizuho-cho, Mizuho-ku, Nagoya 467-8601, Japan; 23 Department of Orthopaedic Surgery, Nihon University Hospital, 1-6 Kanda-Surugadai, Chiyoda-ku, Tokyo 101-8393, Japan; 24 Department of Orthopaedic Surgery, Nihon University School of Medicine, 30-1 Oyaguchi Kami-cho, Itabashi-ku, Tokyo 173-8610, Japan; 25 Department of Orthopedics, Traumatology and Spine Surgery, Kawasaki Medical School, 577, Matsushima, Kurashiki, Okayama 701-0192, Japan; 26 Department of Orthopaedic Surgery, Kitasato University School of Medicine, 1-15-1, Kitazato, Minami-ku, Sagamihara, Kanagawa 252-0374, Japan; 27 Department of Orthopaedic Surgery, Kobe University Graduate School of Medicine, 7-5-1 Kusunoki-cho, Chuo-ku, Kobe 650-0017, Japan; 28 Department of Orthopaedic Surgery, Kochi Medical School, Kochi University, Kohasu, Oko-cho, Nankoku 783-8505, Japan; 29 Department of Orthopaedic Surgery, Graduate School of Medical and Dental Sciences, Kagoshima University, 8-35-1 Sakuragaoka, Kagoshima 890-8520, Japan; 30 Department of Orthopaedic Surgery, Gunma University Graduate School of Medicine, 3-39-22 Showa, Maebashi, Gunma 371-8511, Japan; 31 Department of Orthopaedic Surgery, Mie University Graduate School of Medicine, 2-174 Edobashi, Tsu City, Mie 514-8507, Japan; 32 Department of Orthopaedic Surgery, School of Medicine, International University of Health and Welfare, 852 Hatakeda, Narita, Chiba 286-0124, Japan; 33 Department of Orthopaedic Surgery, International University of Health and Welfare Narita Hospital, 852 Hatakeda, Narita, Chiba 286-0124, Japan; 34 Department of Orthopaedic Surgery and Spine and Spinal Cord Center, International University of Health and Welfare Mita Hospital, 1-4-3 Mita, Minato-ku, Tokyo 108-8329, Japan; 35 Department of Orthopaedic Surgery, The University of Tokyo Hospital, 7-3-1 Hongo, Bunkyo-ku, Tokyo 113-8655, Japan; 36 Department of Orthopaedic Surgery, Osaka University Graduate School of Medicine, 2-2 Yamadaoka, Suita, Osaka 565-0871, Japan; 37 Department of Orthopedics Surgery, Surgical Science, Tokai University School of Medicine, 143 Shimokasuya, Isehara, Kanagawa 259-1193, Japan; 38 Department of Orthopaedic Surgery, Tokyo Medical and Dental University, Yushima 1-5-45, Bunkyo-Ku, Tokyo 113-8519, Japan; 39 Department of Orthopaedic Surgery, University of Yamanashi, 1110 Shimokato, Chuo, Yamanashi 409-3898, Japan; 40 Department of Orthopaedic Surgery, Graduate School of Medicine, Kyoto University, 54 Shogoin-Kawaracho, Sakyo-ku, Kyoto, Kyoto 606-8507, Japan; 41 Department of Orthopaedic Surgery, Faculty of Medicine, University of Toyama, 2630 Sugitani, Toyama, Toyama 930-0194, Japan; 42 Department of Orthopaedic Surgery, Faculty of Medicine, Oita University, 1-1 Idaigaoka, Hasama-machi, Yufu-shi, Oita 879-5593, Japan; 43 Department of Orthopaedic Surgery, Kansai Medical University Hospital, 2-3-1 Shinmachi, Hirakata, Osaka 573-1191, Japan

**Keywords:** delirium, elderly, cervical fracture, cervical spinal cord injury, prediction

## Abstract

The number of elderly patients with cervical trauma is increasing. Such patients are considered to be at high risk for delirium, which is an acute neuropsychological disorder that reduces the patient’s capacity to interact with their environment due to impairments in cognition. This study aimed to establish a risk score that predicts delirium in elderly patients with cervical SCI and/or cervical fracture regardless of treatment type. This retrospective cohort study included 1512 patients aged ≥65 years with cervical SCI and/or cervical fracture. The risk factors for delirium according to treatment type (surgical or conservative) were calculated using multivariate logistic regression. A delirium risk score was established as the simple arithmetic sum of points assigned to variables that were significant in the multivariate analyses. Based on the statistical results, the delirium risk score was defined using six factors: old age (≥80 years), hypoalbuminemia, cervical fracture, major organ injury, dependence on pre-injury mobility, and comorbid diabetes. The score’s area under the curve for the prediction of delirium was 0.66 (*p* < 0.001). Although the current scoring system must be validated with an independent dataset, the system remains beneficial because it can be used after screening examinations upon hospitalization and before deciding the treatment strategy.

## 1. Introduction

Delirium is an acute neuropsychological disorder that reduces the capacity of a patient to interact with their environment due to impairments in cognition [1]. Although the symptoms of delirium are normally reversible, potentially negative effects may persist for both the patient and the healthcare system [2]. These effects include delayed discharge and rehabilitation, an increased risk of adverse events and mortality, and failure to comply with care instructions [3,4]. Although there are treatment options for delirium, including non-pharmacological approaches, the effects of treatment are still limited [2]. Hence, the prevention of delirium is still critical.

The pace of population aging has been accelerating dramatically worldwide. Expectedly, from 2015 to 2050, the proportion of the world’s population aged over 60 years will increase nearly two-fold (from 12% to 22%) [5]. The elderly population experiences high rates of osteoporosis and falls due to declining functional ability [6,7]. Hence, the proportion of individuals aged >60 years with a traumatic spinal cord injury (SCI) has risen from 4.6% in 1970 to 13.2% in 2008 [8]. Moreover, the elderly population is more likely to be diagnosed with a cervical spine injury due to minor trauma, than a thoracic and lumbar spine injury, compared to the younger population [8]. Hence, the number of elderly patients with cervical SCI and/or a cervical fracture has been increasing dramatically [9,10,11].

Old age and life-threatening conditions such as cervical SCI are major risk factors for delirium [12,13]. Consequently, elderly patients with cervical SCI and/or a cervical fracture are considered to be at high risk for delirium. However, due to limitations in healthcare systems, physicians and medical staff cannot always provide intensive preventive measures to all such patients. Therefore, screening tools for delirium in elderly patients with cervical SCI and/or cervical fracture should be established to select patients who are at especially high risk for delirium.

Although there are several screening tools for delirium, standard indicators for delirium screening have not been uniformly recognized [14]. Previously proposed screening tools, such as the Abbreviated Mental Test, the 4 A’s Test, the Brief Confusion Assessment Method, reciting the months of the year backward, and the Single Question in Delirium, are reported to be effective predictors of delirium in geriatric inpatients [15]. However, the use of these tools on admission may not be ideal for patients with cervical SCI and/or cervical fracture, as many of them require interviews that might not be immediately feasible for patients with cervical trauma. Additionally, it is important to take preventive measures for delirium immediately upon admission of the patient. Therefore, the current study aimed to create a screening tool that can predict delirium during treatment (regardless of whether surgical or conservative) in elderly patients with cervical SCI and/or cervical fracture. The current screening tool was designed to predict delirium without any data obtained from lengthy interviews with the patient.

## 2. Materials and Methods

### 2.1. Patient Population

This study analyzed multi-center registry data retrospectively collected by the Japan Association of Spine Surgeons with Ambition (JASA) [16]. Registrars reviewed the medical records and retrospectively registered cases into the JASA database based on the following inclusion and exclusion criteria:

- Inclusion criteria: patients aged ≥65 years with traumatic cervical SCI and/or traumatic cervical fracture; patients treated conservatively or surgically between 2010 and 2020 at an institution registered in the JASA and those who were followed for at least three months after the injury;
- Exclusion criteria: patients with cervical metastasis; and those with any missing data;
- Registrars did not exclude patients on the basis of specific medications, surgical procedures, surgical instruments, and/or reasons other than the inclusion/exclusion criteria indicated above.

In total, 1512 patients from 78 institutions were registered in the JASA database (average age: 75.8 ± 6.9 years; 1007 males and 505 females; 1310 patients were transferred to a hospital within 24 h of injury; 202 patients were hospitalized at an average of 10.0 ± 16.9 days after the injury). All registered patients were included in the current analysis.

**Figure 1:**
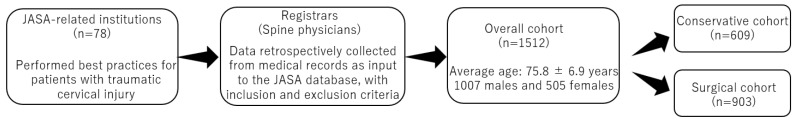
Flowchart of the patient selection and data collection processes. All registered patients were included in the current analysis.

The patients were divided into two cohorts according to treatment type: the conservative cohort (including patients who underwent conservative therapy for traumatic cervical SCI and/or traumatic cervical fracture) and the surgical cohort (including patients who underwent surgery for the injury, whether expected or unexpected) (Figure 1).

### 2.2. Collected Data

All data regarding patient background, delirium status, neurological impairment scale, therapy, and radiography were extracted from our registry database.

#### 2.2.1. Patient Background Data

Data regarding the age at injury, sex, height, weight, body mass index, pre-injury mobility (independent, able to walk with assistance, or wheelchair/bedridden), blood examinations at the first visit, and comorbidities (dementia, diabetes, and hypertension) were collected. Blood tests included the levels of hemoglobin (Hb; g/dL), total protein (g/dL), and albumin (Alb; g/dL). All assessments were performed immediately after patient transfers and hospital visits.

#### 2.2.2. Delirium Data

The existence or absence of delirium during the in-hospital stay was collected from medical records retrospectively. Standard tools such as the Diagnostic and Statistical Manual of Mental Disorders, 4th or 5th Edition, and the Confusion Assessment Method were used to diagnose delirium [2].

#### 2.2.3. Radiographic Data

Collected radiographic data included the presence of a cervical fracture and cervical ossification of the posterior longitudinal ligament (OPLL), as detected by plain radiography and/or computed tomography (CT). Additionally, data regarding signal changes in the spinal cord on T2-weighted magnetic resonance imaging was collected, and comorbid major organ injuries were evaluated using whole-body CT when necessary. All assessments were performed immediately after patient transfers and hospital visits. Major organ injuries were defined as head, chest, abdominal organ, and pelvic injuries.

#### 2.2.4. Neurological Impairment Scale

The American Spinal Cord Injury Association (ASIA) Impairment Scale was used as a parameter of neurological impairment [17]. All assessments were performed immediately after patient transfers and hospital visits.

#### 2.2.5. Therapeutic Data

For the conservative cohort, information was collected regarding the existence of acute steroid use and neck fixation (neck brace or halo-traction). For the surgical cohort, the existence of early surgical intervention within 24 h of injury and surgical type (posterior decompression, posterior fusion ± decompression, anterior fusion ± decompression, or combined fusion ± decompression) were recorded.

### 2.3. Statistical Analysis

All analyses were performed using SPSS software (version 23; SPSS, Chicago, IL, USA). A *p*-value of <0.05 was considered statistically significant.

#### 2.3.1. Risk Factors for Delirium

The incidence of delirium was compared between the conservative and surgical cohorts using the chi-square test. Data on patient background factors, radiographic images, neurological impairment scale grade, and therapeutic options were compared between patients with and without delirium in each cohort using the Mann–Whitney U test or chi-square test, as appropriate. A residual analysis was performed to confirm the chi-square test results for specific cervical alignment parameters. The results of the residual analysis were described as *p* < 0.05 when the variable showed |r| >1.96, in accordance with the Haberman method [18]. For each cohort, significant variables with *p* < 0.05 on univariate analysis were included in a multivariate analysis as explanatory variables; the presence of delirium was set as the objective variable. In the multivariate analysis, continuous or non-binary data were translated into binary values before calculation using previously reported thresholds [19,20,21,22]. The adjusted odds ratio (aOR) and 95% confidence interval (CI) of the dependent variables were calculated.

#### 2.3.2. Delirium Risk Score

A delirium risk score was established as the simple arithmetic sum of points assigned to variables that were significant in the multivariate analyses of the two cohorts. The points assigned to each variable were decided based on the adjusted risk relationship: 1 point for an aOR of 1.0−10 and 2 points for an aOR > 10 [23]. When specific factors were calculated as risk factors of delirium in both cohorts, the factor was treated as a single factor in the final scoring system. The predictive value of the delirium risk score was evaluated by a receiver operating characteristic (ROC) curve analysis using data from the total cohort. The area under the ROC curve (AUC), 95% CI, sensitivity, and specificity were calculated for various score thresholds.

## 3. Results

### 3.1. Patient Characteristics

The conservative cohort included 609 patients (average age: 77.1 ± 7.6 years; 226 patients were aged ≥80 years; 376 males and 233 females; in-hospital stay: 82.8 ± 26.7 days; follow-up period: 17.9 ± 22.1 months). The surgical cohort included 903 patients (average age: 75.0 ± 6.3 years; 233 patients were aged ≥80 years; 631 males and 272 females; in-hospital stay: 74.9 ± 30.5 days; follow-up period: 20.0 ± 20.1 months). Delirium was diagnosed in 56 patients (9.2%) in the conservative cohort and 66 patients (7.3%) in the surgical cohort during the in-hospital stay. There were no significant differences in the incidence of delirium between the conservative and surgical cohorts (*p* = 0.211).

### 3.2. Risk Factors of Delirium in the Conservative Cohort

On univariate analysis in the conservative cohort, there were significant differences in age (*p* < 0.001), pre-injury mobility (*p* = 0.028), Hb (*p* = 0.038), and Alb levels (*p* = 0.011), ASIA Impairment Scale grade (*p* = 0.022), number of patients with cervical fracture (*p* = 0.025), and comorbid major organ injury (*p* = 0.006) between patients with and without delirium (Table 1). After binarizing age, mobility, and Alb and Hb levels using previously published thresholds (age: 80 years, mobility: independent or non-independent, Alb: 3.5 g/dL, Hb: 12 g/dL) [19,20,21], the multivariate analysis revealed old age (≥80 years; aOR: 2.26, *p* = 0.024), hypoalbuminemia (<3.5 g/dL; aOR: 2.15, *p* = 0.043), cervical fracture (aOR: 2.33, *p* = 0.020), and comorbid major organ injury (aOR: 2.01, *p* = 0.045) as independent variables related to the occurrence of delirium in the conservative cohort (Table 2).

### 3.3. Risk Factors of Delirium in the Surgical Cohort

On univariate analysis in the surgical cohort, there were significant differences in age (*p* = 0.003), sex (*p* < 0.001), pre-injury mobility (*p* = 0.024), ASIA Impairment Scale grade (*p* = 0.008), number of patients with dementia (*p* < 0.001), diabetes (*p* = 0.045), cervical fracture (*p* = 0.019), cervical OPLL (*p* = 0.015), and a signal change in the spinal cord (*p* < 0.001) between patients with and without delirium (Table 3). After binarizing age, mobility, and ASIA Impairment Scale grade using previous thresholds (age: 80 years, mobility: independent or non-independent, ASIA Impairment Scale: A, B, or C, D, E) [19,22], the multivariate analysis revealed old age (≥80 years; aOR: 2.75, *p* < 0.001), dependence in pre-injury mobility (aOR: 2.28, *p* = 0.023), comorbid diabetes (aOR: 1.91, *p* = 0.030), and presence of a cervical fracture (aOR: 2.33, *p* = 0.020) as independent variables related to the occurrence of delirium in the surgical cohort (Table 4).

### 3.4. Establishment of a Delirium Risk Score

**Figure 2:**
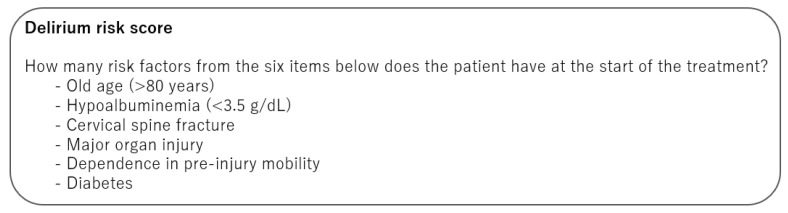
Delirium risk score.

**Figure 3:**
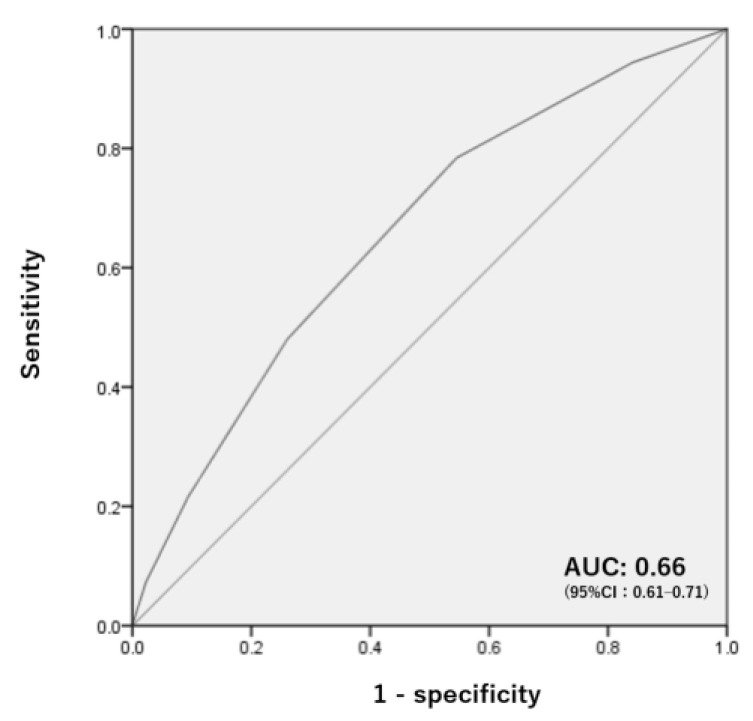
ROC curve analysis of the delirium risk score (n = 1512). The x-axis and y-axis indicate “1-specificity” and “sensitivity”, respectively. The straight line indicates random chance, and the curved line indicates the ROC curve with a greater leftward curve indicating better accuracy. ROC—receiver operating characteristic curve; AUC—area under the curve; CI—confidence interval.

Based on the results of the multivariate analyses of the two cohorts, old age (>80 years), hypoalbuminemia (<3.5 g/dL), dependence in pre-injury mobility, the presence of a cervical spine fracture, comorbid major organ injury, and comorbid diabetes were included in the delirium risk score calculation (Figure 2). Each variable was scored at 1 point based on the calculated aORs and indicated definitions. The delirium risk score was calculated as the sum of the six variables, with a total score varying from 0 to 6. The ROC analysis using data from the total cohort revealed that the AUC of the score for predicting delirium was 0.66 (95% CI: 0.61–0.71, *p* < 0.001, Figure 3). For a risk score threshold of 2 points, the sensitivity was 0.784 and the specificity was 0.455 (Table 5). For a risk score threshold of 3 points, the sensitivity was 0.480 and the specificity was 0.740.

## 4. Discussion

In our dataset, approximately 10% of elderly patients with cervical cord injury and/or cervical fracture who were treated conservatively or surgically developed delirium during the in-hospital stay. We established a screening system for delirium using six risk factors, including older age, hypoalbuminemia, cervical spine fracture, major organ injury, dependence on pre-injury mobility, and diabetes. Patients with at least two of these six risk factors could be predicted to develop delirium during treatment with 78% sensitivity and 46% specificity, regardless of the type of therapy.

There is no consensus on how to establish a scoring system. For example, the Spine Instability Neoplastic Score, which is a standard scoring system for patients with spinal metastasis, was recently created using expert opinions [24]. In contrast, the Katagiri scoring system, which predicts the prognosis of patients with skeletal metastasis, was recently created using the results of statistical tests [25]. In the current study, we created a delirium scoring system by combining risk factors (as determined by regression analysis) from two cohorts. This approach was used because we aimed to create a scoring system that could be applied to elderly patients with traumatic SCI and/or cervical fractures regardless of the selected therapeutic method. Additionally, the current scoring system was developed using cases with cervical SCI as well as cases with cervical fractures, enabling physicians to apply this scoring to patients with cervical fractures who are at risk of neurological deterioration after admission.

Delirium is known to negatively impact both the healthcare system and the patient. Leslie et al. [4] analyzed hospitalized elderly patients in the non-intensive care general medical unit and concluded that patients who experienced delirium during hospitalization had a 62% increased risk of mortality, with an average loss of 13% in life years, compared to that for patients without delirium. Additionally, in a study on the effect of delirium in patients with traumatic SCI by Cheung et al. [13], patients with delirium had a significantly longer hospital stay than the control group. These results indicate the importance of the prevention of delirium as a component of the quality of treatment.

Cheung et al. [13] evaluated the risk factors for delirium in 192 patients with traumatic SCI and concluded that old age at the time of injury and a low initial motor score were risk factors for delirium. Similarly, we found that old age was a risk factor for delirium in both the surgical and conservative treatment cohorts. In addition to old age, we included hypoalbuminemia, dependence on pre-injury mobility, the presence of a cervical fracture, comorbid major organ injury, and comorbid diabetes in our delirium risk scoring system, based on the present statistical results. Dependence on pre-injury mobility might reflect aspects of physical aging that cannot be determined by the number of years of life. Hypoalbuminemia and diabetes are well-known to be associated with delirium [26,27]. The presence of a cervical fracture and a comorbid major organ injury could be considered indicators of an extremely severe trauma, which is reported to be a major risk factor for delirium [28]. Contrarily, the motor score was not identified as a risk factor in either cohort in the current study. The postulated reason for this result is that the cohorts in the current study included patients who suffered a cervical fracture without neurological deficits. Furthermore, sex was not a significant variable in either cohort.

To prevent delirium during in-hospital treatment, the most important non-pharmacological multi-component approaches include (a) attempting to keep patients well-oriented to their surroundings and making them more familiar, (b) providing stimulation to maintain memory and thinking skills, and (c) attempting to improve sleep [29]. Such approaches can reduce the occurrence of delirium by 43% compared to usual hospital care practices [29,30]. Although such treatments show effectiveness in preventing delirium, not all patients are capable of receiving them because of increased medical costs and limitations in medical staffing. Hence, screening tools for the development of delirium can aid physicians and the medical staff by identifying patients potentially at high risk for delirium; the limited medical resources can then be preferentially applied to such patients. We recommend using 2 points as the cut-off value for physicians who apply the current scoring system as a screening tool for delirium to real-world patients evaluated during treatment because of the high sensitivity of the test value. Additionally, we recommend using 3 points as the cut-off value when attempting to identify high-risk patients for delirium because of the high specificity of the test value.

There are several limitations to the present study that need to be addressed. First, the diagnosis of delirium was dependent on the criteria of each institution. Additionally, the timing of the onset of delirium and its severity were not considered. Second, the database used in the current study was already established, and all data were collected retrospectively. This database was imbalanced in terms of sex; there were 1007 males and 505 females. Further, some conditions, such as dementia, might have been under-evaluated, which might have influenced the results of the current study. The data were collected from high-volume trauma centers; patients with severe frailty, dementia, or other degenerative conditions might not have been brought to such institutions. All such differences might bias the current results. Third, this retrospective study analyzed relatively recent data from patients who could be treated with standard delirium measures. Additionally, the retrospective chart review might miss several cases of delirium, especially in those with mild ones. Indeed, the overall incidence of delirium in the current study (8.1%) was low compared to that previously reported [2]. Fourth, our scoring system does not take into account the patient’s psychological perspective. Finally, the AUC of our delirium risk score system was relatively low. Further, the system was not validated in an independent dataset; this constitutes the most considerable limitation of the current study. Accordingly, the current scoring system must be validated in independent samples before clinical application.

The benefit of the current screening tool is that it can be used in patients before treatment decision-making, making it possible to evaluate the delirium risk at an early stage, such as upon admission. Furthermore, the current screening tool can be scored using only data from medical records, without subjective data obtained from interviews by trained experts. Finally, identifying patients at high risk for delirium before deciding on treatment strategies can positively affect the patient’s outcomes.

## 5. Conclusions

The present study establishes a novel delirium risk score for elderly patients with cervical trauma using six risk factors: older age, hypoalbuminemia, dependence on pre-injury mobility, presence of a cervical spine fracture, comorbid major organ injury, and comorbid diabetes. The current screening tool can be applied to patients with cervical SCI and/or cervical fracture using only objective data before decision-making for treatment. Although the current scoring system should be validated in independent, multi-ethnic samples before implementation, its features enable early intervention and the prevention of delirium in the patients who require it the most.

## Figures and Tables

**Table 1:** Univariate analysis in the conservative cohort (n = 609).

	Delirium	Controls	*p*-Value
Number of patients	56	553	
Age (years) ± SD	81.0 ± 7.0	76.7 ± 7.5	<0.001 *
Female/Male	17/39	216/337	0.248 ^#^
BMI ± SD	21.7 ± 5.6	22.0 ± 3.8	0.747 *
Pre-injury mobility			0.028 ^#^
Independent	43	488	
Walk with assistance	12	54	
Wheelchair/bedridden	1	8	
Blood test data			
TP (g/dL) ± SD	6.5 ± 0.9	6.7 ± 0.7	0.165 *
Alb (g/dL) ± SD	3.4 ± 1.0	3.8 ± 0.7	0.011 *
Hb (g/dL) ± SD	12.0 ± 2.2	12.6 ± 1.9	0.038 *
Comorbidity			
Dementia	8	40	0.070 ^#^
Diabetes	11	122	0.738 ^#^
Hypertension	32	267	0.211 ^#^
ASIA Impairment Scale			0.022 ^#^
A/B/C/D/No neurological deficits	6/5/9/12/24	37/20/100/225/171	
Radiographic findings			
Cervical fracture	36	267	0.025 ^#^
Cervical OPLL	10	97	1.000 ^#^
Spinal signal change on MRI	29	287	0.987 ^#^
Comorbid major organs injury	27	167	0.006 ^#^
Conservative therapy			
Steroid use	11	95	0.711 ^#^
Halo-traction	9	57	0.173 ^#^
Neck brace	44	434	0.861 ^#^

* Mann–Whitney U test; ^#^ Chi-square test; SD—standard deviation; BMI—body mass index; TP—total protein; Alb—albumin; Hg—hemoglobin; ASIA—American Spinal Cord Injury Association; OPLL—ossification of posterior longitudinal ligament; MRI—magnetic resonance imaging.

**Table 2:** Multivariate logistic regression in the conservative cohort (n = 609).

Explanatory Variables		Reference	aOR	*p*-Value	95% CI
Age	≥80 years	>80	2.26	0.024	1.11–4.59
Mobility	with assistance	Independent	1.42	0.430	0.59–3.34
Hypoalbuminemia	(3.5 g/dL > Alb)	3.5≤	2.15	0.043	1.03–4.50
Anemia	(12 g/dL > Hb)	12≤	0.77	0.475	0.37–1.59
ASIA scale	A, B	C, D, NoD	1.77	0.204	0.73–4.27
Cervical fracture	with	without	2.33	0.020	1.14–4.76
Major-organ injury	with	without	2.01	0.045	1.01–3.99

aOR—adjusted odds ratio; CI—confidence interval; ASIA: American Spinal Cord Injury Association; ND—no neurological deficits; Alb—Albumin; Hb—hemoglobin.

**Table 3:** Univariate analysis in patients who underwent surgical therapy (n = 903).

	Delirium	Controls	*p*-Value
Number of patients	66	837	
Age (years) ± SD	77.2 ± 6.1	74.8 ± 6.3	0.003 *
Female/Male	12/54	260/577	<0.001 ^#^
BMI ± SD	22.5 ± 3.9	22.1 ± 3.4	0.459 *
Pre-injury mobility			0.024 ^#^
Independent	53	757	
Walk with assistance	6	44	
Wheelchair/bedridden	7	36	
Blood test data			
TP (g/dL) ± SD	6.6 ± 0.7	6.6 ± 0.7	0.825 *
Alb (g/dL) ± SD	3.6 ± 0.6	3.7 ± 0.7	0.241 *
Hb (g/dL) ± SD	12.8 ± 2.0	12.7 ± 1.9	0.712 *
Comorbidity			
Dementia	13	34	<0.001 ^#^
Diabetes	21	176	0.045 ^#^
Hypertension	39	393	0.073 ^#^
ASIA Impairment Scale			0.008 ^#^
A/B/C/D/No neurological deficits	10/3/21/10/22	81/48/196/271/241	
Radiographic findings			
Cervical fracture	48	483	0.019 ^#^
Cervical OPLL	16	109	0.015 ^#^
Spinal signal change on MRI	37	209	<0.001 ^#^
Comorbid major organs injury	18	199	0.550 ^#^
Surgical therapy			
Early intervention (≤24 h)	2	85	0.079 ^#^
Surgical method			
Posterior decomp	14	261	0.094 ^#^
Posterior fusion ± decomp	42	497	
Anterior fusion ± decomp	9	57	
Combined fusion ± decomp	1	22	

* Mann–Whitney U test; ^#^ Chi-square test. SD—standard deviation; BMI—body mass index; TP—total protein; Alb—albumin; Hg—hemoglobin; ASIA—American Spinal Cord Injury Association; OPLL—ossification of posterior longitudinal ligament; MRI—magnetic resonance imaging.

**Table 4:** Multivariate logistic regression in patients who underwent surgical therapy (n = 903).

Explanatory Variables		Reference	aOR	*p*-Value	95% CI
Age	≥80 years	>80	2.75	<0.001	1.58–4.80
Sex	female	male	0.54	0.070	0.28–1.05
Mobility	with assistance	independent	2.28	0.023	1.12–4.62
Diabetes	with	without	1.91	0.030	1.06–3.44
Hypertension	with	without	1.53	0.128	0.89–2.64
ASIA scale	A, B	C, D, ND	1.22	0.604	0.58–2.59
Cervical fracture	with	without	2.15	0.019	1.14–4.06
Cervical OPLL	with	without	0.84	0.567	0.43–1.59
Signal change	with	without	1.08	0.800	0.58–2.02

aOR—adjusted odds ratio; CI—confidence interval; ASIA—American Spinal Cord Injury Association; ND—no neurological deficits; OPLL—ossification of posterior longitudinal ligament.

**Table 5:** Sensitivity and specificity of the delirium risk score (n = 1512).

Number of Factors	Sensitivity	Specificity
1	0.944	0.159
2	0.784	0.455
3	0.480	0.740
4	0.216	0.905
5	0.008	0.992
6	0.001	0.999

## Data Availability

The data that support the findings of this study are available from the corresponding author, K.T., upon reasonable request.
